# Retraction: NOSH-NBP, a novel nitric oxide and hydrogen sulfide- releasing hybrid, attenuates ischemic stroke-induced neuroinflammatory injury by modulating microglia polarization

**DOI:** 10.3389/fncel.2023.1332686

**Published:** 2023-11-24

**Authors:** Frontiers Editorial Office

Following publication, concerns were raised regarding the integrity of the images in the published figures. Image duplication concerns were identified within **Figures 4A and 7A**. The authors failed to provide a satisfactory explanation and the complete raw data during the investigation, which was conducted in accordance with Frontiers' policies. As a result, the data and conclusions of the article have been deemed unreliable and the article has been retracted.

This retraction was approved by the Chief Editor of Frontiers in Cellular Neuroscience and the Chief Executive Editor of Frontiers. The authors did not state whether they agree or disagree with this retraction.

